# Ghrelin's Roles in Stress, Mood, and Anxiety Regulation

**DOI:** 10.1155/2010/460549

**Published:** 2010-02-14

**Authors:** Jen-Chieh Chuang, Jeffrey M. Zigman

**Affiliations:** ^1^Divisions of Hypothalamic Research and Endocrinology & Metabolism, Department of Internal Medicine, The University of Texas, Southwestern Medical Center, 5323 Harry Hines Blvd., Dallas, TX 75390-9077, USA; ^2^Department of Psychiatry, The University of Texas, Southwestern Medical Center, 5323 Harry Hines Blvd., Dallas, TX 75390-9077, USA

## Abstract

Several studies suggest that the peptide hormone ghrelin mediates some of the usual behavioral responses to acute and chronic stress. Circulating ghrelin levels have been found to rise following stress. It has been proposed that this elevated ghrelin helps animals cope with stress by generating antidepressant-like behavioral adaptations, although another study suggests that decreasing CNS ghrelin expression has antidepressant-like effects. Ghrelin also seems to have effects on anxiety, although these have been shown to be alternatively anxiogenic or anxiolytic. The current review discusses our current understanding of ghrelin's roles in stress, mood, and anxiety.

## 1. Introduction

Metabolic syndrome and psychiatric disorders have become leading threats to the public health worldwide, and associations between the two now have been reported in several studies. For instance, a growing body of literature indicates that obesity is an important environmental risk factor for developing affective disorders. As an example, in a large cross sectional epidemiological U.S. study, a body mass index ≥30 was found to be associated with a 25% higher rate of mood disorders [1, 2]. Conversely, other studies suggest that psychological stress can increase the risk of developing obesity. For instance, a longitudinal study found that major depression in late adolescent girls was associated with a 2.3-fold increased risk of obesity in adulthood [3]. Also, a chart review of U.S. veterans with posttraumatic stress disorder showed a significantly increased rate of obesity [4]. Thus, it seems likely that certain circulating hormones and critical neuroanatomical circuits exist that regulate both energy homeostasis and our psychological state. Work from a handful of laboratories now suggests that the peptide hormone ghrelin is one such mediator of both behaviors linked to food intake and body weight and behaviors associated with psychosocial stress, mood, and anxiety.

## 2. Changes in Ghrelin Associated withPsychosocial Stress

**Figure 1:**
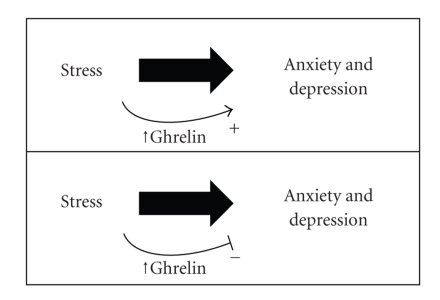
Two opposing models for ghrelin action in the behavioral responses to stress. Published studies all seem to agree that ghrelin levels rise upon stress of various types. Some studies suggest that rising ghrelin would contribute to the mechanisms responsible for the development of stress-induced depression and anxiety (upper panel), while others suggest that rising ghrelin helps minimize what otherwise would be more severe manifestations of depression and anxiety following stress (lower panel).

We and others have found that rises in ghrelin occur not only in response to states of energy insufficiency [5–8] but also following stress [9] (Figure 1). For example, elevations in either gastric ghrelin mRNA or total plasma ghrelin have been observed in response to various models of acute stress, including following a tail pinch stress protocol in ddy mice and following a water avoidance stress protocol in Wistar Kyoto and Sprague-Dawley rat [10, 11]. Also, rises in desacyl and acylated ghrelin plasma levels, preproghrelin mRNA levels, and numbers of ghrelin cells were shown in Wistar rats following 5 days continuous exposure to 2 cm of water [12]. Our own study found that acylated ghrelin levels rise in C57BL6/J mice in response to chronic social defeat stress (CSDS); in particular, ghrelin was significantly elevated on the day following the 10-day CSDS protocol and remained elevated when assessed again one month later [9]. In addition, human subjects subjected acutely to psychosocial stress also display increased plasma ghrelin [13]. Supportively, epinephrine, which increases with stress, can increase circulating ghrelin levels [14].

## 3. Ghrelin's Role in Mood

The effects of these stress-induced increases in ghrelin likely include effects on metabolism-related physiology and behavior as well as effects on mood. Our own work using mouse models has revealed that increasing circulating ghrelin levels by 10 days of calorie restriction or by acute s.c. injection produces antidepressant-like responses in the forced swim test [9]. However, caloric restriction no longer induced these responses in mice lacking ghrelin receptors (GHSR-null mice), thus suggesting that interference with ghrelin signaling negates the antidepressant-like behaviors associated with calorie restriction [9]. Also, upon challenge with the CSDS protocol, GHSR-null mice manifested greater social isolation (another marker of depressive-like behavior) than did wild-type littermates [9]. Thus, it has been suggested that activation of ghrelin signaling pathways in response to chronic stress may be a homeostatic adaptation that helps individuals cope with stress (Figure 1, lower panel). 

 We are aware of only one other study that examines the effects on mood of manipulations to ghrelin expression [15]. For this latter trial, behaviors were examined in rats subsequent to i.c.v. administration of antisense ghrelin oligonucleotides. Rats receiving the antisense ghrelin DNA exhibited much less immobility in the forced swim test as compared to rats receiving scrambled oligonucleotides, thus suggesting an antidepressant-like effect [15]. Associative studies that examine a ghrelin-mood relationship also exist, including one in which a GHSR polymorphism was found associated with major depressive disorder [16] and another in which total plasma ghrelin levels were compared among subjects with major depression, schizophrenia, and controls [17]. 

 It is also important to note that ghrelin now has been shown in a handful of studies to affect reward behavior of various types. For example, ghrelin lowers the threshold dose of cocaine required to establish a conditioned place preference, is required for alcohol reward, and itself can elicit a conditioned place preference [18–20]. Ghrelin also increases neuronal activity in brain reward centers in humans shown images of appealing foods [21] and has been shown to enhance the rewarding value of high-fat diet when administered to *a* *d* *l* *i* *b*-fed mice [22]. These findings are relevant to the discussion of mood as anhedonia is a major component of most forms of depression and as reward behaviors and mood-related behaviors share many of the same neural circuits (see below) [23].

## 4. Ghrelin's Role in Anxiety

Several groups have investigated ghrelin's effects on anxiety-like behaviors. Using models identical to those described above, we showed that increasing circulating ghrelin levels by calorie restricting mice for ten days or by acute s.c. administration of ghrelin to *a* *d* *l* *i* *b*-fed C57BL6/J mice produces anxiolytic-like responses in the elevated plus maze [9]. However, when GHSR-null mice were calorie restricted, no longer were these anxiolytic-like behavioral responses observed [9]. Thus, we proposed that ghrelin has anxiolytic-like effects and that ghrelin signaling is required for the anxiolytic-like effects of caloric restriction [9]. Our observations seem to be supported by a report showing that Wistar Kyoto rats, which are thought to display more anxiety-like behaviors than Sprague-Dawley and other rat strains, have lower plasma levels of ghrelin than Sprague-Dawley rats [24]. Furthermore, although stress-induced elevations in circulating ghrelin have been noted in both Wistar Kyoto and Sprague-Dawley rat strains, the magnitude of those elevations was significantly lower in the anxiety-prone Wistar Kyoto animals than in the Sprague-Dawley animals [11]. 

 These findings of anxiolytic-like effects of raised ghrelin levels differ from the results of several other studies. In one of these studies, i.c.v. or i.p. administration of ghrelin to ddy mice decreased duration of time in and number of entries into the open arms of an elevated plus maze (anxiogenic-like actions) when assessed ten minutes after injection [10]. Another group demonstrated that i.c.v. administration of ghrelin or its direct microinjection into the hippocampus, amygdale, or dorsal raphe nucleus induced anxiety-like behaviors in certain rat strains when assessed 5 minutes later in the elevated plus maze, open field test and step-down/inhibitory avoidance test [25, 26]. Also, i.c.v. administration of antisense ghrelin oligonucleotides induced not only antidepressant-like behaviors but also anxiolytic-like responses in rats [15]. Finally, a recent study demonstrated that i.c.v. administration of ghrelin to chicks can induce anxiogenesis [27]. 

 The reasons for the varied anxiety-related behavioral responses to changes in ghrelin signaling are not clear, at present (Figure 1, upper versus lower panels). They could potentially be due to differences in dose, route of administration, timing of administration, timing of behavioral test after administration, strain or species, or other experimental details such as handling of animals. Strain-dependent differences in performance in various behavioral tasks, such as the elevated plus maze and forced swim test are not uncommon (as an example, in one study, only one out of four inbred strains of mice exhibited sensitivity to fluoxetine in the forced swim test [28]). Further studies will be required to sort out how these discrepant anxiety-related animal study findings for ghrelin translate into behavioral effects in humans. 

## 5. Potential Mechanisms by Which Ghrelin Regulates Mood

The mechanisms by which ghrelin affects mood-related behaviors have not yet been fully elucidated, but likely include interaction with its receptors in one or more brain sites critical to mood determination. We have shown that ghrelin's ability to decrease immobility in the forced swim test is dependent on the presence of orexin [9], and previous work has demonstrated that the antidepressant-like responses to calorie restriction (which also causes an increase in ghrelin) requires orexin [29]. Both direct and indirect links between ghrelin and orexin exist. The most direct link would involve binding of ghrelin to GHSRs present on orexin neurons. Such would be supported by previous studies demonstrating GHSRs within the lateral hypothalamic area of rat, where orexin-containing neuronal cell bodies exist [30], as well as those showing that ghrelin can induce action potentials in isolated orexin neurons [31]. Alternatively, ghrelin might indirectly engage the orexin system by targeting neurons at other locations which, in turn, project to the lateral hypothalamic area. For instance, several studies suggest that ghrelin directly engages its receptors on AgRP/NPY neurons of the hypothalamic arcuate nucleus (as reviewed in [32, 33]), which are also known to project to lateral hypothalamic orexin neurons [34]. 

 Ghrelin's actions on mood also might involve direct interaction with GHSR-containing neurons that exist within the ventral tegmental area. There is growing evidence for a role of the ventral tegmental area (VTA) and its dopaminergic projections to the nucleus accumbens in mood regulation and depression [23]. These circuits, in addition to related projections from the VTA to the amygdala and limbic regions of neocortex, are particularly involved in motivation, the valuation of rewards, the establishment of reward-associated memories and the ability to experience pleasure; impairment of all of these features prominently in the manifestation of depression [35, 36]. As just one example of VTA involvement in depression, chronic social defeat stress has been shown to be associated with a significant increase in VTA dopamine neuron firing rates [37]. In fact, ghrelin also increases action potential frequency in ventral tegmental area neurons and induces dopamine release into the nucleus accumbens [38–40]. Furthermore, ventral tegmental area microinjection of ghrelin increases food intake while microinjection of a GHSR antagonist into the VTA decreases food intake in response to i.p.-injected ghrelin [38, 41]. 

 A large body of work has identified the hippocampus as being involved in antidepressant efficacy and other aspects of depression, including that associated with stress [42]. Many studies have shown that the hippocampus together with the neocortex mediates cognitive aspects of depression such as memory impairment and feelings of worthlessness, hopelessness, guilt, and suicidality [42]. Also, antidepressant therapy stimulates hippocampal neurogenesis, in a time course that seems consistent with the delayed onset of therapeutic action of antidepressant agents [43]. Of interest, GHSRs are known to be expressed within all regions of the hippocampus [30, 44, 45]. In addition, peripherally administered ghrelin is taken up by and increases spine synapse density within the hippocampus [46]. Ghrelin-deficient mice perform poorly in tests of behavioral memory, while ghrelin administration reverses these deficits [46]. Direct microinjection of ghrelin into the hippocampus dose-dependently increases memory retention [26]. Ghrelin also recently has been shown to stimulate cellular proliferation and differentiation of adult rat hippocampal progenitor cells [47, 48], thus suggesting that ghrelin also might induce hippocampal neurogenesis. 

 Finally, ghrelin's action on mood may be mediated through the modulation of brain inflammation. Mounting evidence indicates that inflammation may play a role in psychiatric diseases (as reviewed in [49, 50]). For example, correlative studies have suggested the association between inflammation markers and depressive symptoms [51]. In addition, several studies have shown that administration of cytokine or cytokine inducers such as LPS or vaccine can lead to the development of depressive symptoms, while antiinflammatory therapy generates antidepressant-like effects [52–54]. GHSRs have been found to be expressed in immunocytes [55] and ghrelin or ghrelin mimetics also have been shown to have immunosuppressive actions via the inhibition of proinflammatory cytokines such as IL1-beta, IL-6, and TNF-alpha [56–59]. Together, these data suggest that the stress-induced elevations in ghrelin may help to alleviate the potential damage that could be caused by inflammation within the brain.

## 6. Summary

Several groups have now demonstrated that rises in ghrelin occur not only during periods of energy insufficiency but also following either acute or chronic stress. Investigations into the ramifications of these stress-associated ghrelin increases are only in their early stages. Our own work suggests that these raised ghrelin levels may help to minimize the deleterious, depression-like behaviors often associated with stress, but perhaps at the expense of a worsened metabolic profile. Future studies are needed to sort out ghrelin's effects on anxiety-like behaviors, as these have been shown by different groups to be either anxiogenic or anxiolytic. Certainly, it is crucial that these differences in the proposed action of ghrelin on anxiety-like behaviors be resolved given the impact that they might have on the side-effect profile of any GHSR antagonist in development as an antiobesity or antidiabetes agent. Also, the antidepressant-like actions and possible anxiolytic-like actions of ghrelin potentially might enhance the effectiveness of ghrelin mimetics being considered for the treatment of cachexia or anorexia nervosa. Future studies should also be directed towards determining the mechanisms by which ghrelin acts to have its effects on mood-related and anxiety-related behaviors as well as the pathways responsible for the stress-induced elevations in ghrelin.
